# Cartographic Analysis of Antennas and Towers: A Novel Approach to Improving the Implementation and Data Transmission of mHealth Tools on Mobile Networks

**DOI:** 10.2196/mhealth.3941

**Published:** 2015-06-04

**Authors:** William Brown III, Mobolaji Ibitoye, Suzanne Bakken, Rebecca Schnall, Iván Balán, Timothy Frasca, Alex Carballo-Diéguez

**Affiliations:** ^1^ HIV Center for Clinical and Behavioral Studies New York State Psychiatric Institute Columbia University New York, NY United States; ^2^ College of Physicians and Surgeons Department of Biomedical Informatics Columbia University New York, NY United States; ^3^ Mailman School of Public Health Sociomedical Sciences Columbia University New York, NY United States; ^4^ School of Nursing Columbia University New York, NY United States; ^5^ Columbia University Medical Center Department of Psychiatry Columbia University New York, NY United States

**Keywords:** cartographic analysis, mHealth, mobile health, antenna, short message service, text messaging, SMS, wireless, HIV

## Abstract

**Background:**

Most mHealth tools such as short message service (SMS), mobile apps, wireless pill counters, and ingestible wireless monitors use mobile antennas to communicate. Limited signal availability, often due to poor antenna infrastructure, negatively impacts the implementation of mHealth tools and remote data collection. Assessing the antenna infrastructure prior to starting a study can help mitigate this problem. Currently, there are no studies that detail whether and how the antenna infrastructure of a study site or area is assessed.

**Objective:**

To address this literature gap, we analyze and discuss the use of a cartographic analysis of antennas and towers (CAAT) for mobile communications for geographically assessing mobile antenna and tower infrastructure and identifying signal availability for mobile devices prior to the implementation of an SMS-based mHealth pilot study.

**Methods:**

An alpha test of the SMS system was performed using 11 site staff. A CAAT for the study area’s mobile network was performed after the alpha test and pre-implementation of the pilot study. The pilot study used a convenience sample of 11 high-risk men who have sex with men who were given human immunodeficiency virus test kits for testing nonmonogamous sexual partners before intercourse. Product use and sexual behavior were tracked through SMS. Message frequency analyses were performed on the SMS text messages, and SMS sent/received frequencies of 11 staff and 11 pilot study participants were compared.

**Results:**

The CAAT helped us to successfully identify strengths and weaknesses in mobile service capacity within a 3-mile radius from the epicenters of four New York City boroughs. During the alpha test, before CAAT, 1176/1202 (97.84%) text messages were sent to staff, of which 26/1176 (2.21%) failed. After the CAAT, 2934 messages were sent to pilot study participants and none failed.

**Conclusions:**

The CAAT effectively illustrated the research area’s mobile infrastructure and signal availability, which allowed us to improve study setup and sent message success rates. The SMS messages were sent and received with a lower fail rate than those reported in previous studies.

## Introduction

Short message service (SMS) text messaging is one of the most ubiquitous digital forms of communication in the world—an average of 350 billion text messages a month are sent across the world’s mobile networks [1]. A recent US survey reported that 84.06% (1914/2277, margin of error 3 percentage points) of adults own mobile phones and of those 73.00% (1212/1914) send and receive SMS text messages. Young adults age 18 to 24 lead this trend, exchanging an average of 109.5 messages daily [2]. Due to the ubiquity of mobile communication networks, mHealth—particularly the use of SMS—is growing as an innovative remote data collection and intervention method in biomedical research.

Searches performed on the National Library of Medicine’s MEDLINE/PubMed database indicate that the biomedical literature on SMS nearly doubles each year. Title and abstract searches for the terms ”short message service” and “text messaging” yielded 563 articles published between December 2000 and January 2013 (Table 1). Sorting these results by publication year revealed the yearly growth in the number of publications on these topics, indicating a substantial increase in the implementation of SMS in biomedical research. There was a 43.2% increase in the number of papers in 2010 compared to 2009, a 92.1% increase in 2011 compared to 2010, and a 94.2% increase in 2012 compared to 2011.

Researchers studying the use of remote data collection and transmission in biomedical research have demonstrated the potential for data collection efficiency and accuracy, real-time behavioral reporting, and assisting adherence to biomedical protocols [3-9]. However, multiple systematic literature reviews on SMS applications for disease prevention in developing countries reported several barriers to SMS system use [5,10]. One barrier is mobile network signal fluctuations, often due to tower positioning and obstructions. The second is short signal ranges. Both of these barriers affect feasibility, implementation, and acceptability [5,10-12]. As an example, one study included 9000 youth using SMS for seeking and reporting health information. Of the 2160/9000 (24%) dissatisfied users, 259/2160 (12.00%) did not receive a response to their SMS due to mobile network fluctuations, and 1080/2160 (50.00%) complained about related timing delays in receiving responses [5].

Identifying areas with a greater number of antenna towers and more optimal positioning of antenna towers can address signal fluctuations and short signal ranges. Currently, there are no studies in the literature that detail if and how the mobile infrastructure of a study area was assessed. We sought to fill this gap in the literature by conducting a cartographic analysis of the mobile infrastructure in our area prior to the implementation of a SMS pilot study assessing the utility of SMS text messaging to improve adherence and data collection to a biomedical protocol. Though the pilot study used SMS, the standardized communication protocols used by SMS are identical to those used to transmit data to and from most mHealth tools in biomedical research [8,13-15]. As such, the aim of this paper is to describe the use of a cartographic analysis of antennas and towers (CAAT) for mobile communications for geographically assessing mobile antenna and tower infrastructure and identifying signal availability for mobile devices. The findings from this study are widely applicable to other studies using mHealth tools.

**Table 1:** SMS articles published between December 2000 and January 2013.

Publication year	Number of PubMed publications	Annual increase from previous Year (%)
Dec 2000-Jan 2013	563	—
2012	235	94.2%
2011	121	92.1%
2010	63	43.2%
2009	44	—

## Methods

### SMS System Alpha Test

Before conducting the pilot study, a test team at the research site conducted simulated operational testing. Eleven staff members were asked to test the system and act as alpha test participants (N=11). Each of the eleven staff members/participants reported mock behavior via SMS once a day for one week and answered three to five branched questions per reporting session. Questions varied based on the answers that were given. After participants finished their reporting period, they were prompted for general feedback via email. Participants were asked, open-endedly, to discuss thoughts, ideas, suggestions, and concerns. Topics reported included system speeds, errors, system typos, reporting typos, question structure changes, SMS algorithm changes, reporting session length, and overall acceptability. Descriptive statistics were calculated on SMS text message sent and received data.

Analysis of SMS sent and received data from the alpha test identified several failed message deliveries that were due to weak mobile service signals. A failed message status, as indicated by our open-source SMS system, means the message was unable to be sent to the mobile service provider for delivery. After confirming all hardware and software was functioning correctly, weak mobile service signal was the last possible reason for failed messages. Weak signal was confirmed using the signal strength gauge on a mobile phone with the same service provider. Thus, signal strength was a potential barrier to the successful implementation and collection of the SMS data. To better determine the feasibility of implementing the pilot study in New York City and to identify geographic areas of mobile service signal strength and weakness, we assessed the robustness of antenna tower networks within the research area.

### Cartographic Methods

The research area that was alpha tested for the pilot study was New York City, which has five boroughs and a varied landscape of buildings, foliage, and altitudes. The site and location of our text messaging system was the HIV Center for Clinical and Behavioral studies at the Columbia University Medical Center (CUMC) in the northern region of the borough of Manhattan.

Data collection success depends on both sending messages as well as each participant receiving messages. Therefore, it was important to assess the research area where the participants live and conduct most of their daily activities. Thus, in addition to upper Manhattan, cartographic analyses were performed on lower Manhattan and the other three most populous of the five boroughs of New York City (Brooklyn, Queens, and the Bronx). Each borough was assessed in a 1 to 3 mile radius (depending on borough) of each outer borough’s epicenter. Areas of mobile service signal strengths and weakness were identified, and our implementation strategy for the subsequent pilot study was modified accordingly.

### Pilot Study

This pilot study was approved by the New York State Psychiatric Institute-Columbia University Department of Psychiatry Institutional Review Board. The pilot study drew its biomedical protocol and study population from a parent study “HIV Home Test and Decision-Making Among HIV-Negative Men" [16]. Eleven participants from the parent study were recontacted and enrolled in the pilot study. Study participants comprised an ethnically diverse sample of men who reported frequently engaging in unprotected anal intercourse with nonmonogamous male partners. All men were New York City residents at the time of enrollment, male, 18 years of age or older, fluent in English, human immunodeficiency virus (HIV)-negative (confirmed at enrollment), and aware that unprotected anal intercourse (UAI) with a partner of HIV-positive or unknown serostatus carries risk of HIV transmission.

Participants were then enrolled in the study and given 12 OraQuick Rapid HIV-1/2 test kits, with the option to use them to test their nonmonogamous sexual partners and/or themselves before intercourse over the course of the 10-week study period. Condoms were provided with each test kit.

### Pilot Study SMS Procedures

Participants were trained to use an SMS system to report their HIV test kit use. Participants reported their sexual behavior and HIV test kit use daily via SMS. They were also asked to specify which time of the day they preferred to receive text messages reminding them to send their report into the system. After the first 3-week period of HIV test kit use and SMS reporting, participants were asked to stop reporting for one month so that researchers could do a preliminary system assessment. During this month participants were allowed to continue HIV test kit use. After the 1-month period, participants were asked to continue reporting to the system for another 3-week period. In case of a positive HIV test result or other problems, participants could contact study staff via an emergency hotline or SMS text message.

One dollar in compensation was provided for each SMS report completed, plus an additional $25 to offset participant SMS sending and receiving costs. The maximum possible compensation per participant was $165.

### Open Source SMS System

We used FrontlineSMS to send and receive text messages. FrontlineSMS was chosen based on its versatility and breadth of system features. The SMS system consisted of four components: (1) an open source computer-based application where researchers logged in and managed sending and receiving messages, contact information, user preferences, and passcodes; (2) a message database; (3) a module to set message reminder frequency; and (4) a global system for mobile communications (GSM) modem to send and receive text messages.

To ensure the privacy of study participants, messages were processed through a third-party SMS gateway carrier with message encryption capability. All sent and received messages were encrypted. In addition, the service contract included an agreement that the messages processed on their servers would be encrypted and protected and that privacy would be maintained. At the study site a record of all the messages was stored on a password-protected server in a secured location.

### Social Network Environmental Scan of GSM Modems

The GSM modem provides the direct link from the computer to the GSM network. FrontlineSMS provides a detailed list of tested GSM modems (and phones that can be used as GSM modems) that are compatible with FrontlineSMS’s open-source software [17]. An environmental scan was performed within FrontlineSMS’s user social network community on GSM modem use, which helped to designate the most effective modem to use for the study. We systematically searched the social network community forum discussion threads with the following keywords: modem, United States, works, GSM, price, Falcom, Huawei, Wavecom, Sierra Compass, Onda, ZTE Incorporated, Samba, E Series, Fastrack, 885, MC8781, mini modem, and MF627. After aggregating search results and analyzing the success rates and functionalities described in the forum, we chose the Huawei E Series GSM modem because it had the best reported performance and most positive responses, and we purchased it from a non-FrontlineSMS vendor.

### Cartographic Analysis of Antennas and Towers

We conducted our cartographic analysis to develop a geographic map of the study area’s antennas and towers and to obtain detailed service provider information. We used AntennaSearch, which provides information on service providers, antennas, and both existing and future towers, to facilitate the analysis. Antennas are the actual emitters of radio signals. Antennas can be placed on towers (multiple) or can be installed to stand alone on top of existing buildings. Stand-alone antennas are usually small (under 200 ft). It is also possible to check multiple antennas to determine which cell phone carriers are located on a particular tower. Existing towers can be registered or nonregistered structures where antennas are placed. Towers may be used for various services including cellular, paging, and microwave. Future towers indicate newly filed (or pending) applications to construct new towers. Application information includes location coordinates and detailed ownership data [18].

Our cartographic analysis of antennas and towers consisted of sorting mobile carriers by geographic proximity, distance imputation, and general urban topology. These methods were applied to detect geographic mobile service changes, identify resources, analyze proximities, and assess the general robustness of the study area’s mobile service infrastructure. This involved identifying an address location as the true epicenter of the area to be surveyed and imputing that address into a web-based mobile service tower search and mapping engine. The data collected included information on service ownership, antenna and tower locations, distance from the specified site, owner contact information for antennas and existing towers, and projections for the development of future towers.

### SMS Message Frequency Analysis

We calculated descriptive statistics of the SMS messages. For the data analysis, we analyzed all messages in both the alpha test phase and pilot study. Alpha test and SMS pilot data were analyzed together, disaggregated, and categorically. Messages were sorted first by message type (pending, sent, received, failed), then by date and time, and finally by message content.

## Results

### Results From Cartographic Analysis of Antennas and Towers

**Figure 1:**
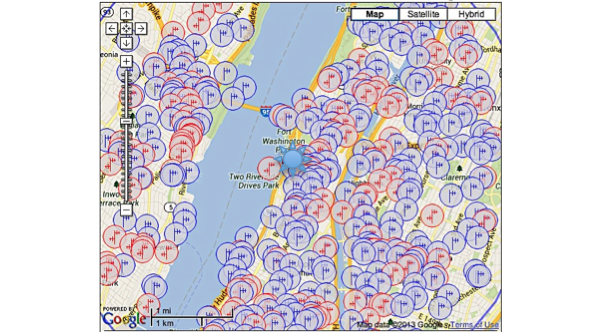
Antenna sites: 1050 Riverside Dr, New York, NY 10032. [Magnification 1]. Blue/single antennas indicate a small (below 100ft) stand-alone antenna on top of a structure, and a red/double antenna indicates multiple antennas sharing a high tower structure.

**Figure 2:**
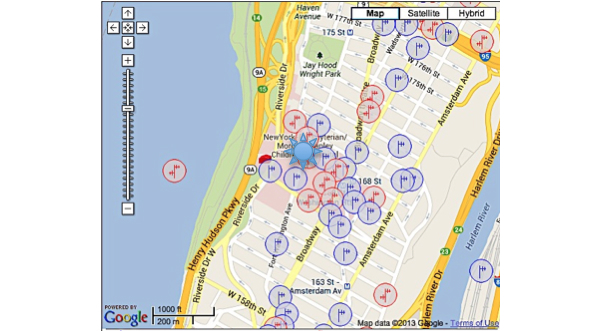
Antenna sites: 1050 Riverside Dr, New York, NY 10032. [Magnification 2].

**Figure 3:**
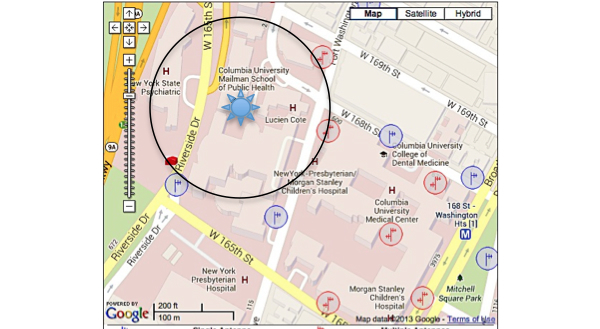
Antenna sites: 1050 Riverside Dr, New York, NY 10032. [Magnification 3].

One hundred and sixteen towers (3 registered, 113 not registered) were found within three miles of our study site; in addition, applications for three future towers were found. There were a total of 965 antennas found. Though the general area was robust and had a significant amount of towers within a mile of the study site, the building that the study was housed in was located in a service gap (Figures 1-3). For the alpha testing phase of implementation, messages were serviced by T-Mobile USA, Inc. The cartographic analysis showed service providers within a three-mile radius of the study site and the number of antennas they owned. At the time of our analysis, Nextel Communications, Inc. was the largest commercial service provider, followed by Clearwire Spectrum Holdings, Northrop Grumman Systems Corp, and ATT Corp (Table 2). There were no antenna towers privately owned and serviced by T-Mobile. Nextel had no text-message-only service plans and required a contract beyond the life of the study, which would not have been cost effective. Therefore, for the actual pilot study we switched to ATT Corp for its robustness and signal strength at the study site as determined by the cartographic analysis.

The images below demonstrate the breadth of service provided in the cartographic analysis. Zoomed images provide a magnified view of service gaps that may not be evident at lower magnifications and broader geographic views. Blue/single antennas indicate a small (below 100ft) stand-alone antenna on top of a structure, and a red/double antenna indicates multiple antennas sharing a high tower structure. Black circles indicate gaps in service—areas where mobile service antennas are either not present or distant.

**Table 2:** Service providers and antennas in the study area.

Service providers	Number of antennas
City Of New York	58
Nextel of New York Inc.	57
New York City Police Department	56
Horizon Communications	33
Clearwire Spectrum Holdings III LLC	31
Northrop Grumman Systems Corp	28
New York City Transit Authority	19
ATT Corp	13
FCI 900 Inc.	12
New York City of Manhattan	12

**Figure 4:**
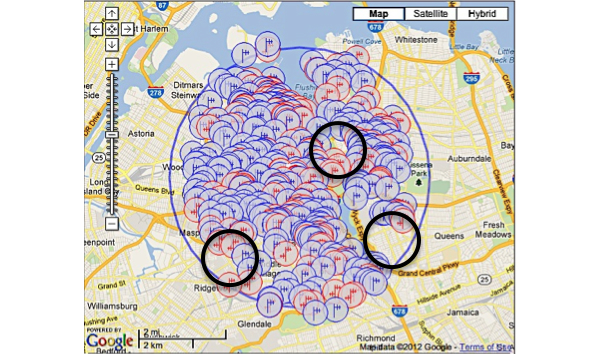
Antenna sites: Queens Borough, New York. Black circles indicate gaps in service.

**Figure 5:**
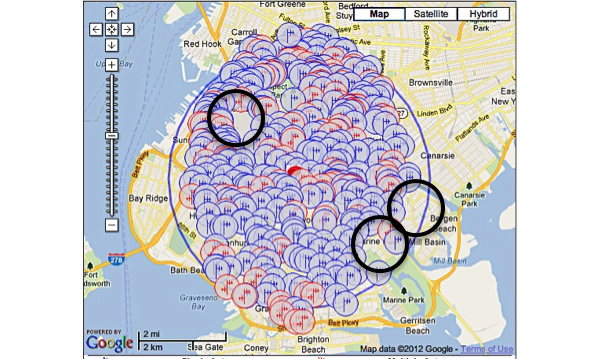
Antenna sites: Brooklyn Borough, New York. Black circles indicate gaps in service.

**Figure 6:**
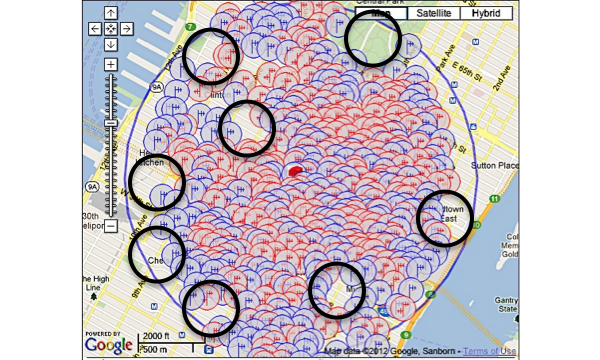
Antenna sites: Manhattan Borough, New York. Black circles indicate gaps in service.

**Figure 7:**
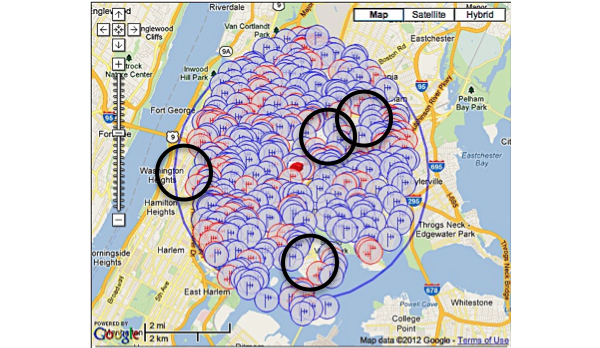
Antenna sites: Bronx Borough, New York. Black circles indicate gaps in service.

In Queens, there were 242 towers (4 registered, 238 not registered), 3 new tower applications, and 762 antennas detected (Figure 4). In Brooklyn, there were 121 towers (3 registered, 118 not registered), 3 new tower applications, and 648 antennas detected (Figure 5). The study site was located in upper Manhattan. Thus, we assessed the mobile service topology for upper Manhattan around the study site (Figures 1-3). In lower Manhattan, the radius of analysis was reduced to one mile to compensate for its narrow latitude. There were 183 tower structures (8 registered, 175 not registered), 7 new tower applications, and 2618 antenna locations detected (Figure 6). In the Bronx there were 105 tower structures (5 registered, 100 not registered), 2 new tower applications, and 805 antenna locations detected (Figure 7). All four boroughs and sites qualified as high volume signal areas and were deemed robust enough to support the study needs.

Unlike the cartographic analysis of the study site, the cartographic analysis of the boroughs only demonstrates analyses and views of large geographic areas (Figures 4-7). This was done because there was no specific location from which participants were expected to always send and receive messages. Thus, a general assessment of the area was most appropriate and provided enough content and context to assess the availability of mobile signal to study participants.

### Results From SMS Message Frequency Analysis

We downloaded data from FrontlineSMS as a .csv file and organized the data in Microsoft Excel. For the SMS message frequency analysis we separated the data into two main categories, message type and message status, which work together to characterize a performed action. Message type describes the kind of message and is divided into two subcategories, sent and received. Message status describes the state or action of the message and is divided into four subcategories: sent, received, pending, and failed. Sent, pending, or failed message statuses are only applied to sent message types. The received message status is only applied to the received message type. A sent message status means the message was successfully transmitted to the mobile service provider for delivery to participants. Pending means the message is still being processed by the program’s algorithm and/or the message has yet to be sent to the mobile service provider. A failed message status means the message was unable to be sent to the mobile service provider for delivery to the participant. A received message status means the system successfully received the participant’s message from the mobile service provider.

Over the course of the alpha test and the pilot study there were a total of 6223 messages, excluding miscellaneous messages used for pinging or programming the system (see Table 3). Of the 6223 messages, 4136 (66.46%) were sent messages that originated from the FrontlineSMS program and 2087 (33.53%) were received messages that originated from alpha test or pilot study participants. Of the 4136 messages sent, 4109 (99.35%) were sent successfully, 26 (0.63%) failed to be sent, and 0 were pending or unprocessed. Of the 2087 messages received, all were received properly with no message processing errors.

**Table 3:** Comparison of the alpha test and pilot study SMS data.

SMS messages	Alpha test (%)	Pilot study (%)	Total (%)
All sent	1202/1817 (66.15)	2934/4406 (66.59)	4136/6223 (66.46)
All received	615/1817 (33.85)	1472/4406 (33.41)	2087/6223 (33.54)
Successful sent	1176/1202 (97.84)	2934/2934 (100.00)	4109/4136 (99.35)
Successful received	615/615 (100.00)	1472/1472 (100.00)	2087/2087 (100.00)
Failed sent	26/1202 (2.16)	0	26/4136 (0.63)
Failed received	0	0	0
Total	1817	4406	6223

Comparison of the alpha test and pilot study data showed system improvement in successful data transmission. Of the 1817 messages processed for the alpha test, there were 1202 (66.15%) messages sent to participants and 615 (33.85%) received from participants. Of the total sent messages, 1176 (97.84%) had a sent status and 26 (2.16%) had a failed status, with 0 pending. There were 615 received messages and all had a received status. In contrast, 4406 messages were processed in the pilot study. Of those, 2934 (66.59%) were sent and 1472 (33.41%) were received. All sent messages had a sent message status, and all received messages had a received message status, which means all 4406 messages (100.00%) were processed successfully. Thus, after performing a cartographic analysis for antennas and towers we were able to choose the optimal service provider in the area, resulting in zero failed messages.

## Discussion

### Synthesis and Findings

The use of SMS text messaging in health research is growing exponentially, as is the number and diversity of mHealth tools. As mobile technology becomes a staple of everyday life, researchers, practitioners, and interventionists continue to find ways to leverage these technologies to improve the way we advance the health sciences. Since the state of health science and by proxy the state of public health depends on the information collected from those served, then speed, accuracy, and completeness become imperative.

The goal of the SMS pilot study was to understand the feasibility and acceptability of using SMS technology to improve data collection, participant behavior reporting, and adherence to biomedical protocols in research studies. Signal fluctuations and short signal ranges are major challenges to implementing SMS text messaging and other mHealth tools as research technologies. Though the literature on applying SMS technology to research is growing, no studies have scientifically and systematically addressed this challenge until now. We have demonstrated that signal fluctuations and short signal ranges can be mitigated by performing a cartographic analysis to identify areas with a greater number of antennas and towers where the mHealth technology will be used.

Assessing the mobile service infrastructure of the study site and research areas was fundamental to optimizing both data transmission and collection. After performing the cartographic analysis of antennas and towers, we modified our SMS pilot study protocol to take advantage of areas with stronger service and greater numbers of antennas and towers by locating our open-source SMS system in an area with greater signal strength and choosing the provider that had the most service antennas in our research areas. Since areas with greater numbers of antennas and towers provided greater signal strength, we saw an increase in the number of messages that were successfully sent and received. Other factors precipitated by the service provider or participant can have an impact on sent and received success rates. Thus, it was not possible to determine a causal or statistically significant relationship between adjusting the service provider according to the results of the cartographic analysis and the sent/received success rates. However, our analysis indicates a strong correlation between the two. These results elucidate the need and usefulness of performing a cartographic analysis prior to implementing mHealth technology in a research study.

Limitations in mobile service infrastructure could have prevented some messages from being sent and associated messages from being returned during our pilot study. This could also have prevented the behavioral reminders from going out on time, impacting participants’ adherence to study protocol. The cartographic analysis not only informed us of whether our participants and study site would have enough signal availability to send and receive data, but also helped us to choose the optimal service providers in the area. Understanding the service availability of a geographic area is also important for international studies, particularly those in rural and/or less industrialized locations.

During the alpha test the SMS system’s messages were serviced by T-Mobile USA Inc. The cartographic analysis showed that Nextel was the largest commercial service provider in the three-mile radius of the study site, followed by Clearwire Spectrum Holdings, Northrop Grumman Systems Corp, and ATT Corp, respectively. There were no antenna towers privately owned and serviced by T-Mobile. Thus, there was a greater chance for our T-Mobile service to have issues. Moreover, message send and receive data suggested there was a problem with service availability. Nextel had the most antennas in the area and is operated by Sprint Corp. However, at the time of the study, Sprint/Nextel had no text-message-only service plans and required a contract beyond the life of the study. Ultimately, using Sprint/Nextel would not have been cost effective. As a result, we switched to ATT Corp, which was determined by the cartographic analysis to have the second largest number of antennas in the area from a brand name carrier. It also had a cost effective, terminable text-message-only service plan.

Another important outcome of this study is the importance of alpha-testing mHealth tools before implementing them in research. Pilot testing can lead to the identification of bugs in the system that may negatively impact the feasibility and acceptability of a system and allow for troubleshooting prior to deployment. Our alpha test identified study-planning issues that would have a direct impact on our ability to get complete data and may have negatively impacted the experience of the participants. Issues included elements of sentence construction in the SMS message text and comprehension issues. Most importantly, the SMS sent and received data from the alpha test helped us discern that there were mobile service issues. Conducting an alpha test of the system not only helped to identify issues but helped us develop and test solutions to address those issues.

### Limitations and Future Work

AntennaSearch, the information resource used for the cartographic analysis for antennas and towers, only reports data on US states and territories and thus is a limitation for translating these results to future international studies. For researchers wishing to use this technique internationally, cartographic analysis can also be performed using comparable digital resources or paper-based mobile service maps. Though our CAAT depicts antennas with assumed distances and assumed identical capabilities, other factors may affect signal strength, including power of the transmitter or the user cellular phone, antenna positioning, high use periods, and refraction and absorption by buildings and other structures. Thus, it is recommended that researchers be mindful of the information provided by cartographic resources and choose a cartographic resource that best fits their studies’ mobile system and geographic needs.

Moreover, no power analysis was conducted due to the small pool of potential candidates from the parent study (n=27 from “HIV Home Test and Decision-Making Among HIV-Negative Men"), as well as the budgetary constraints of our pilot funds, which only allowed for enrollment of a fraction of the parent study’s participants. Thus, the pilot study was not powered for statistical significance, limiting the generalizability of the findings. Nevertheless, the cartographic analysis demonstrated in this study is unique in implementation and method of application. Future work should include a larger sample size and power calculation and identifying alternative resources for cartographic analysis outside of the US. This method should also be retested in research studies with other mHealth tools. Lastly, our CAAT was performed in New York City, a heavily urban environment. AntennaSearch is likely to yield different results in a rural setting due to differences in elevation and natural and manmade barriers.

### Conclusions

As the literature in health research is beginning to demonstrate, SMS is quickly growing as a desired tool for real-time data collection and monitoring of biomedical adherence in research protocols. Since SMS works on a lowest common denominator mobile technology, it has potential to reach more people and to be user friendly when conducting research internationally, with marginalized populations, or across different languages. If properly implemented and scaled, SMS provides efficient data collection and reliable surrogate markers of adherence to assess viability, safety, acceptability, or efficacy of new biomedical and behavioral interventions.

This study contributes to the growing literature on mHealth use in research and informs the development and improvement of mHealth data collection and adherence tools for biomedical research. The significance of this paper rests in its use of innovative analytics, specifically the use of a cartographic analysis to assess antennas and towers for mobile service availability at our study site and in our broader study area. Comparison of the alpha test and pilot study data show the utility of this type of cartographic analysis in improving SMS system success rates for sent messages. Due to the vast geographic reach of mHealth studies, as well as the large amount of data transmission events, it is imperative to assess mobile service infrastructure and robustness for mHealth studies. The results from this study suggest that cartographic analysis of antennas and towers for mobile service can be used to improve study planning and implementation for mHealth research studies using mobile tools.

## Appendix

Multimedia Appendix 1 SMS text message programming script.

## Abbreviations

CAAT: cartographic analysis of antennas and towers
CUMC: Columbia University Medical Center
SMS: short message service
NLM: National Library of Medicine
HIV: human immunodeficiency virus
UAI: unprotected anal intercourse
GSM: global system for mobile communications
