# Supplementary material for: Cartographic Analysis of Antennas and Towers: A Novel Approach to Improving the Implementation and Data Transmission of mHealth Tools on Mobile Networks
Source: JMIR Mhealth Uhealth. 2015 Jun 4;3(2):e63. doi: 10.2196/mhealth.3941 (PMC4526907; doi:10.2196/mhealth.3941)
Supplement: Multimedia Appendix 1 [file mhealth_v3i2e63_app1.pdf]

# Short Message Service (SMS) Session Question Tree

(SMS Keyword Trigger Style)

P = Participant text, S = SMS system text, [ ] = Action

**P:** Join

**S [add participant]:** Thank you for joining. You will receive a daily reminder to start your SMS session. Questions and answer choices may vary depending on your response

Message speed varies by phone type and/or service provider. Text "help" for more options or "opt out" to quit.

**S [scheduled reminder]:** Are you available for an SMS session? If so, text your password now. If not, text "no"

> **P:** no
>
> **S:** When you are ready text your password to start your session. A reminder will be sent within 24hrs. Thank you.

**P:** (password)

**S [start session]:** Answer by texting the letter & number code (ex: "2b"). You can add text (ex: "2d I lost the kit"), but don't add punctuation or symbols

Text "Help" for options

**S:** 1: Since my last SMS session, I used the HT kit to_

- 1a: test my sex partner
- 1b: test myself
- 1c: test both
- 1d: I did not use an HT kit [skips to Q3]

2: HIV test results showed_

- 2a: no one was positive
- 2b: he was positive [available depending on answer to Q1, notifies researcher]
- 2c: I was positive [available depending on answer to Q1, notifies researcher]

2d: Other (you can enter “2d” and an explanation)

3: Since my last SMS session, I was the_

3a: bottom

3b: top

3c: bottom and top

3d: had no anal sex [skip to Q5]

4: During this sexual encounter a condom was used_

4a: all of the time [end session]

4b: part of the time [end session]

4c: never [end session]

5: Since my last SMS session I discussed the HT kit with a_

5a: potential sex partner [end session]

5b: friend [end session]

5c: no one [end session]

**S [end session]:** Thank you! $1 added. (Optional) Use answer code "6a" to text us anything you think is important (for example: "6a we had alcohol before sex"). You can text about partners, HT kits, drugs, violence, SMS problems, etc.

**To protect your privacy, please delete this SMS session and/or lock your phone.**
[ADDITIONAL COMMENTS/END SESSION]

**S [additional comments/end session]:** Thank you for providing additional comments.
**To protect your privacy, please delete this SMS session and/or lock your phone.**

**P:** opt-out / opt out / optout

**S [remove participant]:** Thank you for being in our study. Please text us why you dropped out.

**P:** Help
**S:** Texting_

“results”= How to interpret test results
“answer” = How to answer SMS questions
“hotline” = Study contact info

“hivinfo” = Info on HIV and local testing

**P:** results
S: One line “-” means negative
Two lines “=” mean positive

**P:** answer
**S:** Answer by texting the letter and number code (ex: "1b"). When adding text, add a space after the code (ex: "1b lost the kit"). Don't add punctuation or symbols.

**P:** hotline
**S:** 24-HOUR CONTACT TO REACH STAFF: 347-467-1009
More info: http://bit.ly/yk8snR or 212-643-6825

**P:** hivinfo
**S:** NY State AIDS Hotline: 1-800-541-AIDS
SIDA Hotline (Spanish): 1-800-233-7432
NYC AIDS Hotline: 1-800-TALK-HIV (1-800-825-5448)

Free HIV testing (20min Results)
AIDS Service Center NYC
41 East 11th Street, 5th Floor, New York, NY 10003
212-645-0875

**P:** <none key word>
**S:** RESPONSE ERROR! Check answer code (no punctuation/symbols) and re-send. If sending a long comment for answer code "6a" ignore this error message.
